# Palmitoleic acid drives gentamicin influx to outpace aminoglycoside-modifying enzymes and overcome resistance

**DOI:** 10.1038/s44259-026-00243-2

**Published:** 2026-07-14

**Authors:** Jamie D. Liu, Iris M. McClain, James R. Manning, Emily N. Kennedy, Hannah R. Friedman, Yury Desyaterik, Elias P. Rosen, Sarah E. Rowe

**Affiliations:** 1 Department of Microbiology and Immunology, University of North Carolina at Chapel Hill, Chapel Hill, NC USA; 2 Eshelman School of Pharmacy, University of North Carolina, Chapel Hill, NC USA

**Keywords:** Drug discovery, Microbiology

## Abstract

Rising antibiotic resistance causes >700,000 deaths annually and could reach ~10 million by 2050. Because the development of new antibiotics lags behind resistance, strategies that restore the activity of approved drugs are urgently needed. Gentamicin efficacy against *Staphylococcus aureus* and *Enterococcus spp*. is limited by widespread aminoglycoside-modifying enzymes (AMEs). Here, we show that palmitoleic acid, a host-derived monounsaturated fatty acid, re-sensitizes resistant gram-positive pathogens to gentamicin by accelerating intracellular drug influx rather than inhibiting AMEs. Single-cell flow cytometry and high-resolution mass spectrometry demonstrate rapid gentamicin accumulation that outpaces enzymatic inactivation. The combination lowers MICs across clinical isolates and significantly enhances killing of mature biofilms at clinically relevant concentrations. These findings establish a framework in which resistance reflects the balance between antibiotic entry and enzymatic inactivation. By shifting this balance toward drug influx, resistance can be functionally reversed without targeting resistance genes, supporting permeability-based adjuvant strategies to expand the utility of existing antibiotics.

## Introduction

Antibiotic resistance is outpacing the development of new antibiotics^1–3^. Compared to drugs administered for life (e.g., for heart disease, diabetes), the profitability of antibiotic development is very low^4^. Consequently, only two new classes of antibiotics have been approved in almost two decades^5^. By 2050, deaths from antibiotic resistant infections are expected to surpass those caused by cancer^6,7^. Therefore, there is an urgent need to reclaim our existing antibiotics by reversing antibiotic resistance and improving antibiotic performance during infection. In this study, we explore the mechanism of using an adjuvant to reverse high-level gentamicin resistance.

Gentamicin is a broad-spectrum, bactericidal aminoglycoside antibiotic that binds to the 30s ribosomal subunit of 16s rRNA and corrupts translation. Mistranslated, truncated proteins are inserted into the cytoplasmic membrane, causing damage, facilitating further gentamicin uptake and bacterial death^8,9^. Gentamicin is commonly used and effective against gram-negative bacteria, including *Escherichia coli*, *Klebsiella pneumoniae*, *Serratia spp*., *Enterobacter spp*., and *Pseudomonas aeruginosa*^10^. Although gentamicin has activity against gram-positive *Staphylococcus aureus* and *Enterococcus spp*., its clinical use is largely limited due to widespread high-level resistance^10–12^. Gentamicin resistance is primarily mediated by aminoglycoside modifying enzymes (AMEs), which include N-acetyltransferases, O-nucleotidyltransferases, and O-phosphotransferases that inactivate gentamicin by the addition of an acetyl group, adenosine monophosphate, or phosphate, respectively^8,9^. These modifications ultimately prevent gentamicin binding to the ribosome. Bifunctional AMEs are capable of multiple types of modification and are transferred via mobile genetic elements (plasmids, integrons, transposons), typically along with other resistance genes^8^. For example, AAC(6’)-Ie-APH(2”)-Ia, the most clinically relevant bifunctional AME, was first identified in *Staphylococcus* and was transferred to *Enterococcus* via horizonal gene transfer^13^. Additionally, AAC(6’)-Ie-APH(2”)-Ia confers high-level resistance to nearly all clinically relevant aminoglycosides^14–16^. It is also common for isolates to contain more than one AME^16^. AAC(6’)-Ie-APH(2”)-Ia is found in 79% of *Enterococcus* clinical isolates, 76% of methicillin-resistant *S. aureus* (MRSA) isolates, and 50% of methicillin-susceptible *S. aureus* (MSSA) isolates^16,17^.

We previously showed that palmitoleic acid potentiates gentamicin killing against gram-positive bacteria, including gentamicin-resistant strains, through an unknown mechanism^18^. Palmitoleic acid is a non-toxic host-derived 16-carbon monounsaturated fatty acid that is synthesized by the liver and adipose tissue and is then incorporated into phospholipids, triglycerides, cholesterol esters, and waxes^19^. In this study, we examine how palmitoleic acid mitigates gentamicin resistance in *Staphylococcus* and *Enterococcus* biofilms.

## Results

### Palmitoleic acid sensitizes resistant gram-positive pathogens to gentamicin

**Fig. 1:**
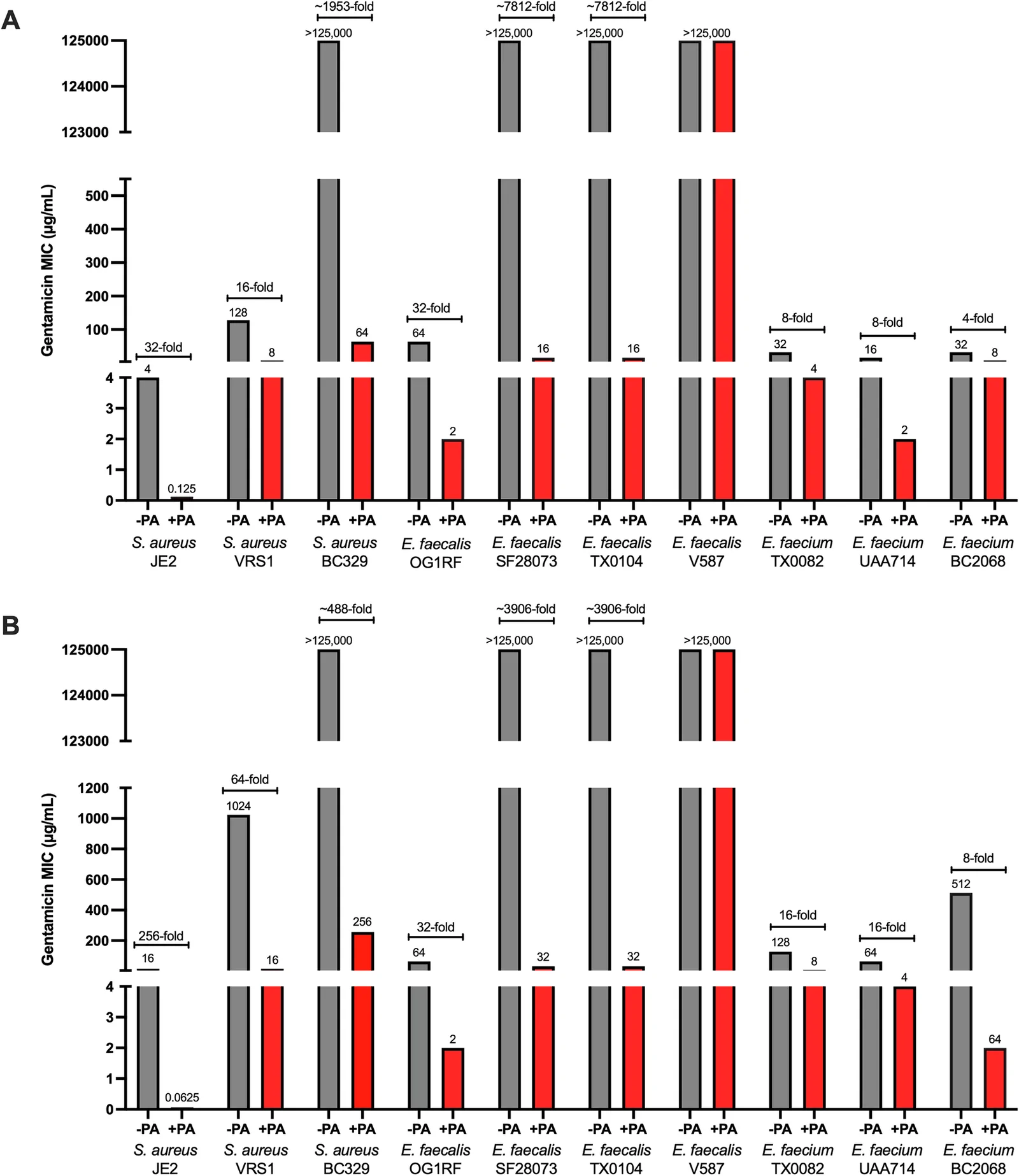
Palmitoleic acid reduces gentamicin resistance in gram-positive bacteria. Minimum inhibitory concentrations (MICs) of gentamicin ± 15 µg/mL palmitoleic acid (PA) against a panel of nine resistant isolates in (**A**) Mueller Hinton Broth (MHB) (**B**) and Brain Heart Infusion (BHI). *S. aureus* strain JE2 was used as a gentamicin-sensitive control. Fold reductions of MICs in the presence of palmitoleic acid are indicated. Data are representative of MICs performed on ≥3 independent days.

Building on our prior observation that palmitoleic acid lowers gentamicin resistance by an unknown mechanism^18^, we assembled a nine-strain panel of gentamicin-resistant isolates; two MRSA, and seven vancomycin-resistant *Enterococcus* (VRE) strains (four *E. faecalis*, and three *E. faecium*). Exposure to sub-inhibitory concentrations of palmitoleic acid (15 µg/mL, Supplementary Table 1), gentamicin minimum inhibitory concentrations (MICs) decreased in eight of nine gentamicin-resistant strains (4- to 7182-fold; Fig. 1A). Notably, highly resistant *E. faecalis* SF28073 and TX0104 (baseline MICs >125 mg/mL) dropped to <1 mg/mL, comparable to concentrations in topical gentamicin formulations used to treat wound infections^10^. For *E. faecalis* V587, the MIC exceeded our assay’s solubility limit (>125 mg/mL), so a palmitoleic acid-mediated reduction may exist but remained beyond measurement (Fig. 1A).

We determined MICs in both Mueller-Hinton broth (MHB; standard for antibiotic susceptibility testing) and Brain Heart Infusion (BHI; which better supports in vitro biofilm formation)^20^. Gentamicin MICs were higher in BHI than in MHB (Fig. 1B), yet 15 µg/mL palmitoleic acid still reduced MICs for nine of ten strains. Thus, even in a nutrient-rich medium, palmitoleic acid broadly sensitizes gentamicin-resistant isolates, supporting this combination as a promising therapeutic approach.

**Fig. 2:**
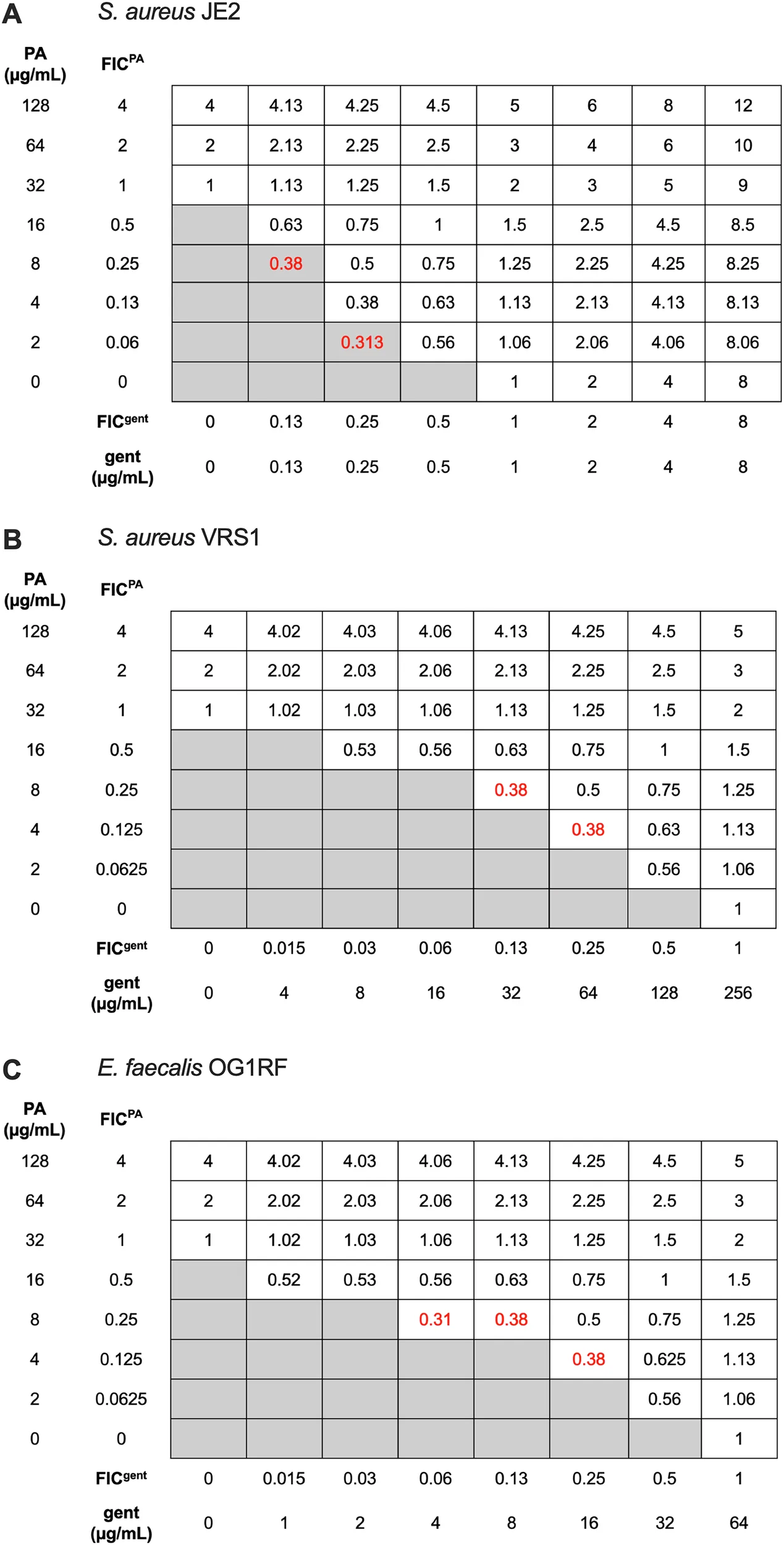
Checkerboard assays demonstrate synergy between palmitoleic acid and gentamicin against gentamicin-resistant strains. **A** *S. aureus* JE2, **B** *S. aureus* VRS1, and **C** *E. faecalis* OG1RF were incubated with increasing concentrations of gentamicin (gent) and palmitoleic acid (PA). OD_600_ was measured. White wells indicate no growth, and gray wells indicate growth. For wells with no growth, the fractional inhibitory concentration (FIC) index was calculated. FICI values ≤ 0.5 are in red text and indicate synergy. Experiments were independently repeated on ≥2 separate days with similar results.

Checkerboard assays on a subset of strains; *S. aureus* VRS1 and *E. faecalis* OG1RF as representative gentamicin-resistant strains and *S. aureus* JE2 as a gentamicin-susceptible control (Fig. 2 and Supplementary Fig. 1) demonstrate synergy between gentamicin and palmitoleic acid. To assess whether this effect extends to other aminoglycosides, we tested kanamycin and amikacin and observed similar potentiation by palmitoleic acid (Supplementary Table 1). Consistent with this, we previously demonstrated palmitoleic-mediated potentiation of gentamicin across a broader range of gram-positive pathogens^18^, supporting the generalizability of this approach.

### Palmitoleic acid sensitizes resistant biofilms to gentamicin

**Fig. 3:**
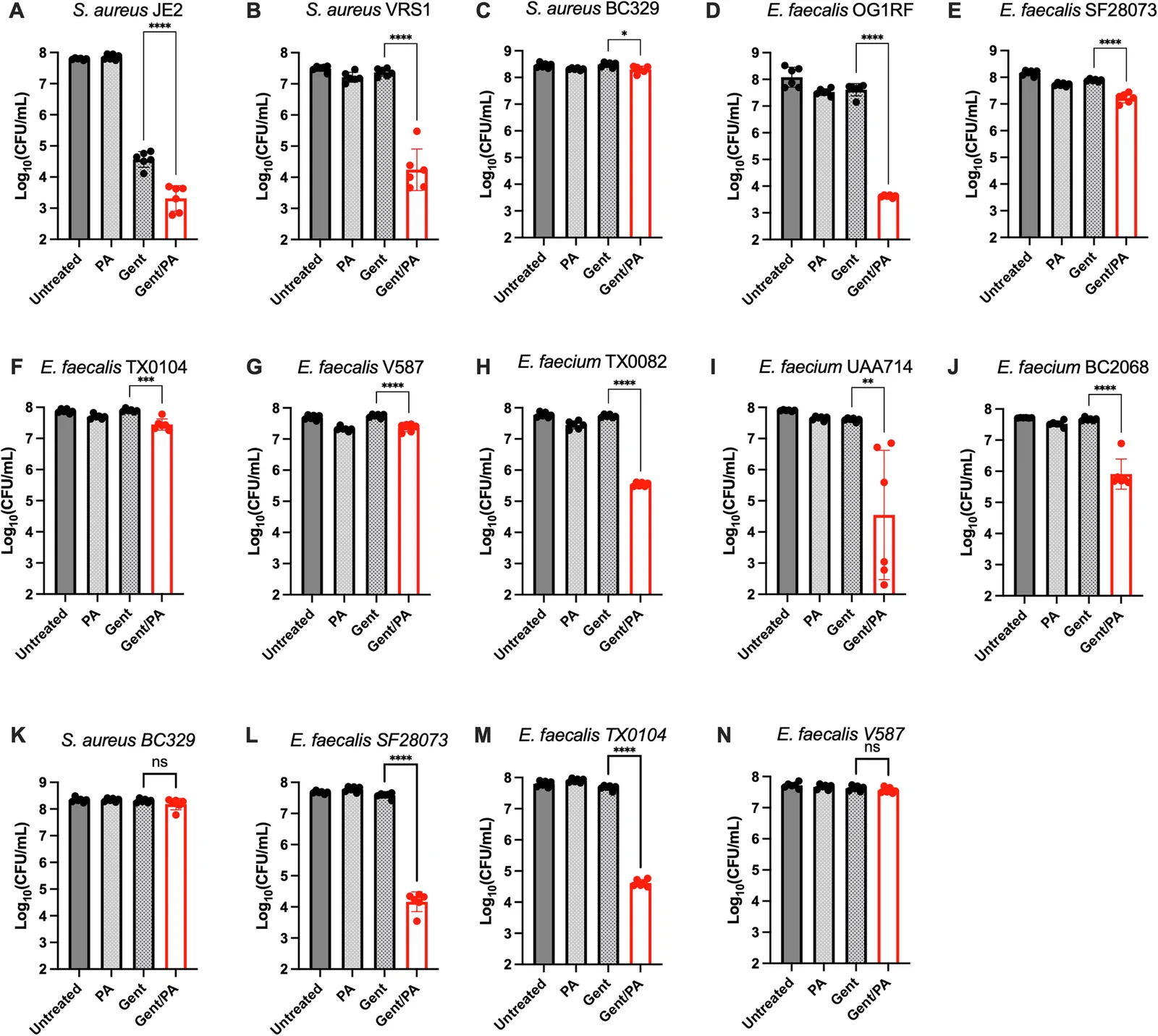
Palmitoleic acid sensitizes highly resistant biofilms to gentamicin. Mature biofilms were cultured for 24 h in BHI media prior to treatment with **A**–**J** 100 µg/mL gentamicin (gent) and/or 30 µg/mL palmitoleic acid (PA) or **K**–**N** 1000 µg/mL gentamicin and/or 30 µg/mL palmitoleic acid. Survivors were enumerated after 24 h. *S. aureus* strain JE2 was used as a gentamicin-sensitive control. Bar graphs show means ± SD from *n* = 6 biological replicates in a representative experiment; experiments were independently repeated on ≥2 separate days with similar results. Statistical significance was determined using an unpaired t-test. ns, *, **, ***, and **** denotes not significant, *p* < 0.05, *p* < 0.01, *p* < 0.001, and *p* < 0.0001 respectively.

A major barrier to successful treatment of bacterial infections is the presence of biofilms, which are implicated in 80% of bacterial infections^21–23^. Biofilms are communities of bacteria that form on biotic and abiotic surfaces and are encased in an extracellular matrix composed of proteins, eDNA, and polysaccharides that protect residing bacteria from host cell assaults and drug treatment^24,25^. To test whether the observed MIC effects translate to biofilms, we challenged mature biofilms with a single-dose regimen across all isolates, 100 µg/mL gentamicin and/or 30 µg/mL palmitoleic acid, even for strains whose MICs exceed 100 µg/mL. Despite this high-level resistance, palmitoleic acid plus gentamicin significantly reduced biofilm burden in all isolates compared to gentamicin alone (Fig. 3 and Table 1). However, the magnitude of killing varied across strains, ranging from up to ~5-log reductions in some isolates (*E. faecalis* OG1RF) to marginal effects in *S. aureus* BC329 and *E. faecalis* V587, where, despite statistical significance, the biological impact is likely limited.

**Table 1:** Biofilm burden reduction of gentamicin and palmitoleic acid treatment

Strain	Percent killing of bacteria	Log reduction
*S. aureus* JE2	99.998	4.952
*S. aureus* VRS1	99.803	3.129
*S. aureus* BC329	28.844	0.184
*E. faecalis* OG1RF	99.999	5.074
*E. faecium* TX0082	99.354	2.195
*E. faecalis* SF28073	76.870	0.665
*E. faecalis* TX0104	62.931	0.451
*E. faecium* UAA714	93.938	3.060
*E. faecalis* V587	57.508	0.378
*E. faecium* BC2068	96.367	1.761

To further evaluate this variability, we selected *S. aureus* BC329 and *E. faecalis* SF28073 and TX0104 strains with the highest baseline gentamicin MICs and the largest MIC reductions in the presence of palmitoleic acid and more marginal reductions in biofilm burden, for testing at higher gentamicin concentrations (1 mg/mL), consistent with levels achieved in topical formulations^10^ (Fig. 3K–M). Under these conditions, enhanced potentiation (~3-logs) was observed in both *E. faecalis* strains, indicating that increased antibiotic exposure can improve potentiation in these backgrounds. In contrast, *S. aureus* BC329 remained refractory to potentiation even at higher gentamicin concentrations, suggesting strain-specific resistance mechanisms may limit the effectiveness of this approach. BC329 is a cystic fibrosis isolate^18^, and patients with cystic fibrosis are frequently treated with inhaled aminoglycosides (e.g., tobramycin), which can reach mg/mL concentrations in airway secretions^26,27^. Thus, prior exposure to high local aminoglycoside levels may have selected for adaptations that reduce susceptibility, although this remains to be determined. In *E. faecalis* V587, increasing gentamicin to 1 mg/mL did not enhance killing with palmitoleic acid compared to gentamicin alone, consistent with the lack of change in gentamicin MIC observed for this strain (Fig. 3N).

For selected strains, gentamicin and/or palmitoleic acid were added directly to established 24-h biofilms without media replacement to assess activity under nutrient-limited conditions. Under these conditions, potentiation was retained in *S. aureus* JE2 and *E. faecalis* OG1RF, although at reduced magnitude, and was not observed in VRS1 (Supplementary Fig. 2A–C). The reduced effect in spent media may be a result of diminished translation, which is required for aminoglycoside-mediated killing^28^. While fresh media may support translation and amplify killing, nutrient-limited conditions likely capture aspects of in vivo biofilms. However, because in vivo environments are dynamic, with fluctuating nutrient availability and waste removal, both conditions may reflect different features of infection. Pre-exposure of biofilms to sub-inhibitory palmitoleic acid (~0.25x MIC) prior to treatment abrogated subsequent potentiation of gentamicin in *S. aureus* VRS1, but not *E. faecalis* OG1RF (Supplementary Fig. 3A, B), indicating that prior fatty acid exposure can alter responsiveness to the combination, indicating that prior fatty acid exposure can alter responsiveness to the combination in a strain-dependent manner and suggesting species-specific adaptive responses.

### Palmitoleic acid induces gentamicin uptake in resistant strains

Gentamicin enters bacterial cell via the proton-motive force (PMF), then binds the 30S ribosomal subunit to corrupt translation and kill the cell^29^. We previously showed that palmitoleic acid increases membrane permeability, accelerating gentamicin entry into susceptible cells^18,30^. In resistant strains, aminoglycoside-modifying enzymes (AMEs) inactivate gentamicin before it reaches the ribosome. Because palmitoleic acid also enhances killing in highly resistant isolates, we hypothesize that palmitoleic acid drives a rapid influx of gentamicin that outpaces AME activity, allowing sufficient drug to reach the ribosome and restore bactericidal activity.

**Fig. 4:**
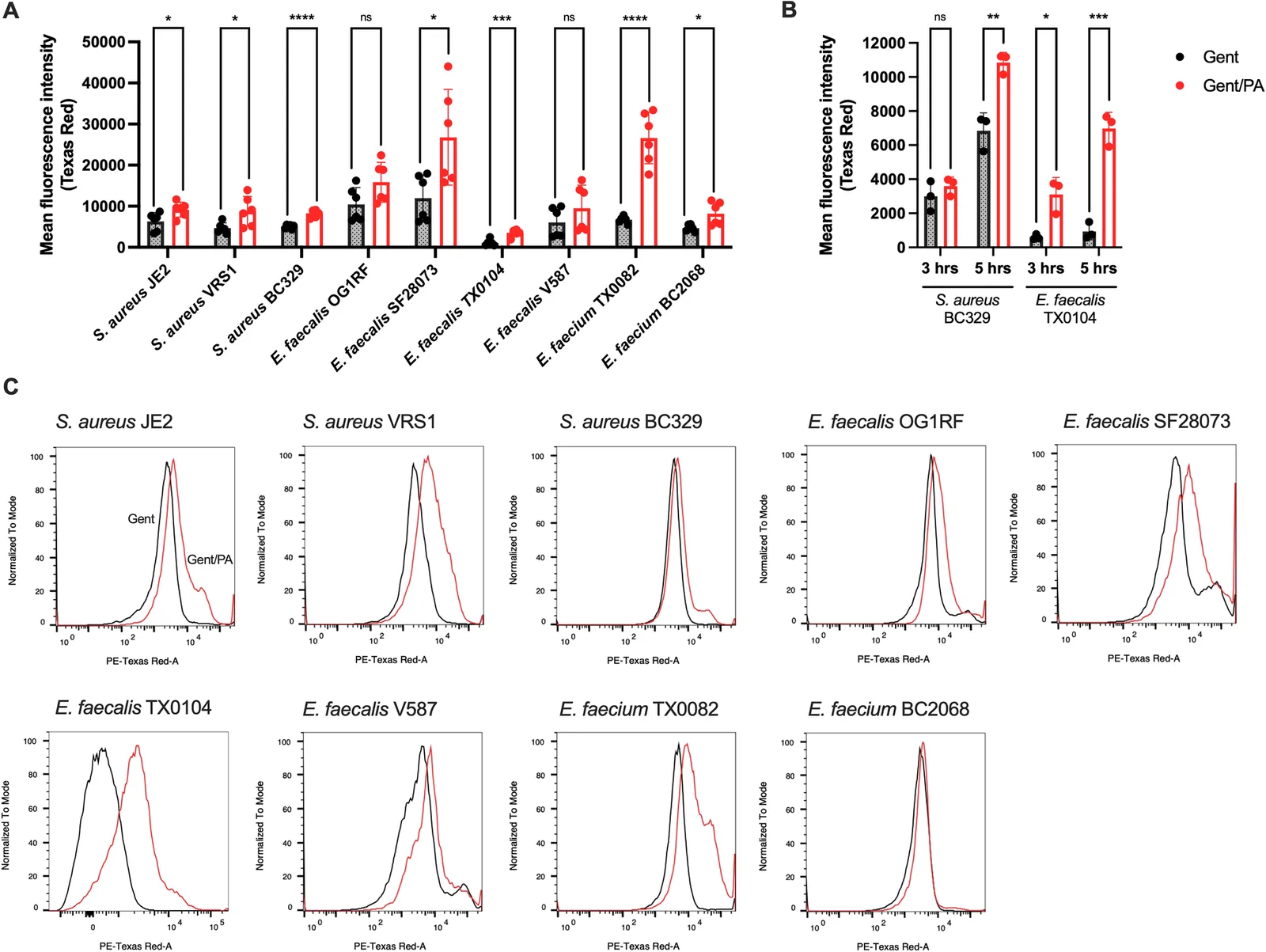
Palmitoleic acid increases gentamicin uptake. **A** Biofilms treated with 100 µg/mL gentamicin (gent) conjugated to Texas Red ± 30 µg/mL palmitoleic acid (PA) for 3 h or **B** 5 h. For **A**, bar graphs show means ± SD from *n* = 6 biological replicates across independent experiments. In **B**, bar graphs show means ± SD from *n* = 3 biological replicates; experiments were independently repeated on ≥2 separate days with similar results. Statistical significance was determined by unpaired t-tests. ns, *, **, ***, and **** denotes not significant, *p* < 0.05, *p* < 0.01, *p* < 0.001, and *p* < 0.0001, respectively. **C** Representative fluorescence histograms of all *S. aureus*, *E. faecalis*, and *E. faecium* strains.

To test whether palmitoleic acid increases gentamicin uptake in resistant strains, we treated mature biofilms with Texas Red-conjugated gentamicin ± palmitoleic acid, dispersed the biofilms, and quantified intracellular gentamicin by flow cytometry. The panel spanned a range of gentamicin susceptibilities: highly resistant (*S. aureus* BC329; *E. faecalis* SF28073, TX0104, V587), intermediate (*S. aureus* VRS1; *E. faecalis* OG1RF; *E. faecium* TX0082, BC2068), and the susceptible control (*S. aureus* JE2). Across all isolates, palmitoleic acid co-treatment increased gentamicin uptake relative to gentamicin alone, indicating enhanced drug entry (Fig. 4A). However, we did not observe a simple relationship between resistance, biofilm killing, and gentamicin uptake (Figs. 1–4). This may, in part, reflect differences in assay timing: uptake was initially measured at 3 h to avoid extensive cell lysis, which limits recovery of intact cells for flow cytometry. Earlier time points were explored; however, intracellular gentamicin levels were frequently below detection limits, precluding reliable comparison between gentamicin monotherapy and gentamicin plus palmitoleic acid.

In highly resistant strains *S. aureus* BC329 and *E. faecalis* TX0104 that showed limited gentamicin uptake in the presence of palmitoleic acid at 3 h, we extended these measurements to later time points (5 h post-treatment). Under these conditions, we observed increased intracellular gentamicin accumulation in both strains (Fig. 4B), indicating that uptake continues over time and may help explain why these strains exhibit large reductions in MIC in the presence of palmitoleic acid (Fig. 1) despite minimal differences in uptake at 3 h (Fig. 4A). Notably, increased uptake at later time points did not uniformly translate to potentiation, as both strains were only marginally potentiated by palmitoleic acid at 100 µg/mL gentamicin (Fig. 3C, F). These differences likely reflect strain-specific variation in aminoglycoside-modifying enzyme expression or regulation.

### AMEs remain active during palmitoleic acid and gentamicin combination treatment

In gentamicin-resistant bacteria, AMEs inactivate gentamicin prior to ribosomal engagement. Based on our uptake and biofilm killing data, we hypothesized that palmitoleic acid does not inhibit AME activity but instead alters the balance between gentamicin entry and enzymatic inactivation, allowing a fraction of incoming drug to remain active. To test whether AMEs remain active during palmitoleic acid-gentamicin treatment, we analyzed the intracellular chemical fate of gentamicin using high-resolution mass spectrometry (HRMS).

**Fig. 5:**
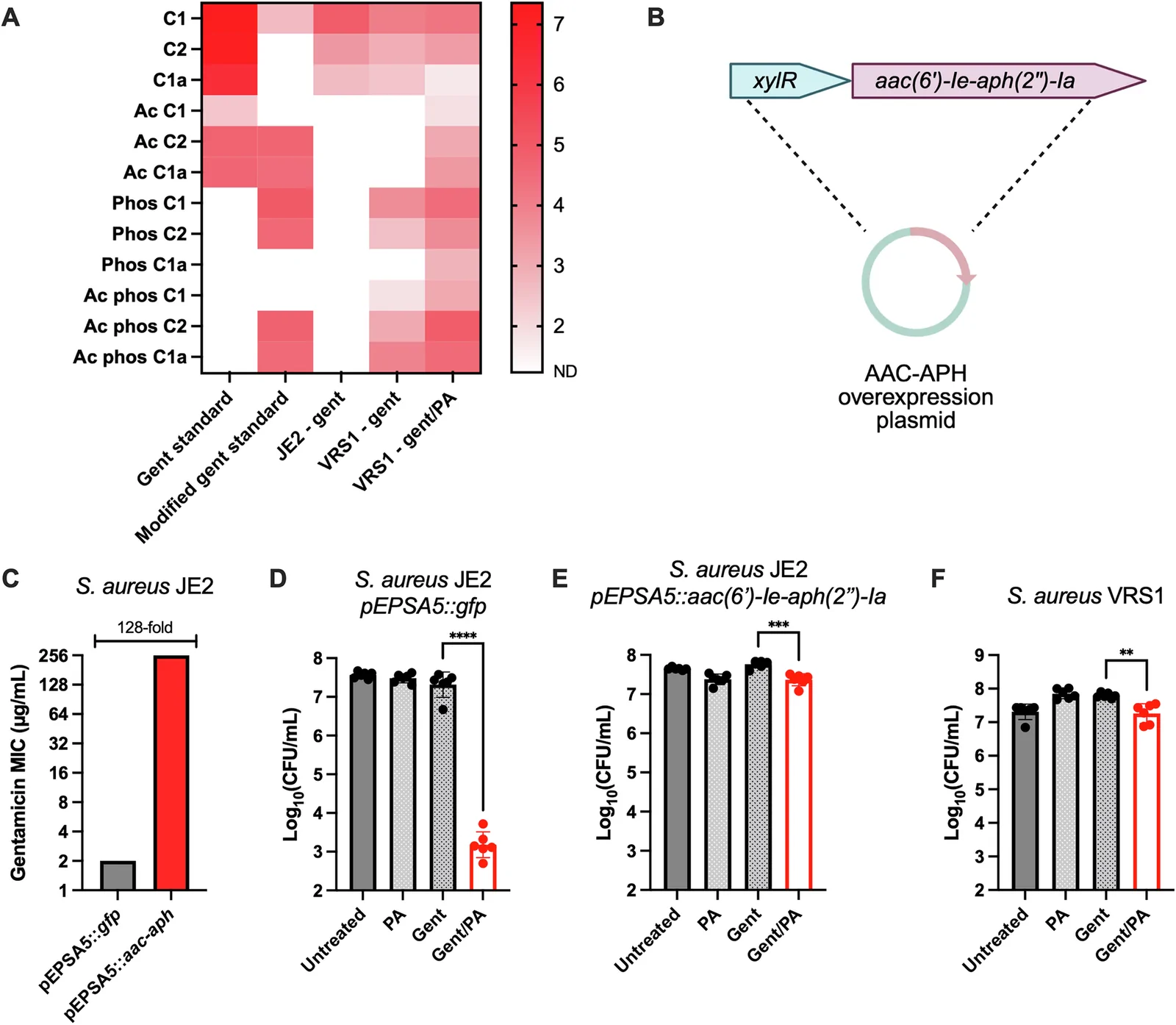
Palmitoleic acid potentiates gentamicin activity by outpacing resistance gene expression. **A** Intracellular chemical fate of gentamicin resolved using high-resolution mass spectrometry (HRMS); Log-normalized signal abundance of gentamicin detected in the commercial active standard (gent) and AME-modified gentamicin in a cell free assay (modified gent), *S. aureus* strains were exposed to 5x MIC gent (JE2 treated with 20 µg/mL and *S. aureus* VRS1 treated with 640 µg/mL gentamicin) ±15 µg/mL palmitoleic acid for 5 h. Ac C1, Ac C1a, Ac C2 denotes acetylated gentamicin; Phos C1, Phos C1a, Phos C2 denotes phosphorylated gentamicin; Ac phos C1, Ac phos C1a, Ac phos C2 denotes phosphorylated and acetylated gentamicin. ND denotes signal abundance that was not detected. **B** Schematic of *pEPSA5* plasmid for xylose-inducible overexpression of *aac(6’)-Ie-aph(2”)-Ia*. **C** The gentamicin MICs of *S. aureus* JE2 harboring *pEPSA5::gfp* or *pEPSA5::aac(6’)-Ie-aph(2”)-Ia*. Biofilm of *S. aureus* JE2 harboring **D** *pEPSA5::gfp* or **E** *pEPSA5::aac(6’)-Ie-aph(2”)-Ia* were treated with 100 µg/mL gentamicin (gent) ± 30 µg/mL palmitoleic acid (PA). **F** Biofilm of *S. aureus* VRS1 that was grown in the presence of 0.25x MIC (32 µg/mL) for 24 h prior to treatment with 100 µg/mL gentamicin ± 30 µg/mL palmitoleic acid. Bar graphs show means ± SD from *n* = 6 biological replicates in a representative experiment; experiments were independently repeated on ≥2 separate days with similar results. Statistical significance was determined using an unpaired t-test. **, ***, and **** denotes *p* < 0.01, *p* < 0.001, and *p* < 0.0001, respectively.

Gentamicin is a heterogeneous antibiotic composed primarily of three species (C1, C1a, and C2), each of which can be enzymatically modified by AMEs through acetylation and/or phosphorylation. HRMS enables direct detection of both active and modified gentamicin species within complex biological samples. Low-level acetylated gentamicin species were present in the commercial gentamicin standard; in contrast, phosphorylated or dual acetylated-phosphorylated species were not detected, indicating that detection of these forms in bacterial samples reflects enzymatic modification by AMEs (Fig. 5A).

In gentamicin-sensitive *S. aureus* JE2, which lacks AMEs, intracellular gentamicin consisted predominantly of active C1 and C2 species following treatment, consistent with unimpaired drug activity. In contrast, the gentamicin-resistant strain *S. aureus* VRS1 contained a mixture of active and enzymatically modified gentamicin species both in the presence and absence of palmitoleic acid, directly demonstrating that AMEs remain active during combination treatment. Notably, phosphorylated and dual acetylated-phosphorylated gentamicin species were detected exclusively in resistant bacterial samples, confirming that gentamicin modification occurs intracellularly in an AME-dependent manner (Fig. 5A and Supplementary Table 2). Palmitoleic acid-gentamicin co-treatment increased overall intracellular gentamicin abundance, including both modified and unmodified species, with a marginal increase in unmodified forms relative to gentamicin alone; these semi-quantitative data are consistent with enhanced drug influx rather than inhibition of enzymatic modification. Because HRMS demonstrates ongoing intracellular gentamicin modification during combination treatment, we next asked whether increasing AME abundance prior to antibiotic exposure would eliminate palmitoleic acid-mediated gentamicin potentiation.

### Palmitoleic acid-induced gentamicin influx outpaces the capacity of the AME

To test whether pre-induction of an AME would limit palmitoleic acid-mediated gentamicin killing, we introduced a plasmid encoding the bifunctional AME AAC(6’)-Ie-APH(2”)-Ia under an inducible promoter into gentamicin-sensitive *S. aureus* JE2 (Fig. 5B). Expression of AAC(6’)-Ie-APH(2”)-Ia increased the gentamicin MIC of JE2 by 128-fold compared to the control (Fig. 5C), confirming robust expression of the resistance enzyme. In mature biofilms, *S. aureus* JE2, which lacks AMEs, was highly susceptible to the gentamicin-palmitoleic acid combination, exhibiting ~4-log reduction in bacterial burden (Fig. 5D). In contrast, pre-induction of AAC(6’)-Ie-APH(2”)-Ia prior to antibiotic exposure markedly reduced killing, limiting bacterial reduction to approximately 0.5 log CFU (Fig. 5E). Thus, while some killing persists, pre-expression of the AME substantially blunts palmitoleic acid-mediated gentamicin potentiation. These data demonstrate that the efficacy of the combination critically depends on a temporal window in which gentamicin influx can outpace AME-mediated gentamicin inactivation. To further test whether the balance between gentamicin influx and enzymatic inactivation governs potentiation, we pre-exposed the resistant strain *S. aureus* VRS1 to sub-inhibitory gentamicin to induce AME expression prior to treatment. Under these conditions, the palmitoleic acid-gentamicin combination no longer enhanced killing in *S. aureus* VRS1 (Fig. 5F), indicating that pre-existing AME activity is sufficient to inactivate incoming drug and prevent potentiation. These findings support a kinetic model in which potentiation depends on a temporal window where gentamicin influx exceeds the capacity of resistance enzymes.

## Discussion

Since the 1980s, there has been a significant gap in antibiotic discovery, with only two new classes of antibiotics identified during this period^31–34^. The antibiotic discovery void, combined with the rapid rise of resistance, has demonstrated the need for revitalizing current antibiotics. Antibiotic adjuvants can be used to reverse resistance and increase treatment efficacy^35,36^. In this study, we found that palmitoleic acid is a potent adjuvant that restores gentamicin efficacy against biofilms of highly resistant strains by driving a surge in intracellular gentamicin that outpaces AME-mediated drug inactivation. Our findings illustrate how manipulating drug influx can be leveraged to restore the activity of existing antibiotics. A similar influx-inactivation balance operates in gram-negative pathogens. For carbapenems, outer membrane porins (e.g., OmpK35/OmpK36 in *K. pneumoniae* or OprD in *P. aeruginosa*) are the main entry routes into the periplasm^37,38^. Porin loss, downregulation, or channel narrowing reduces carbapenem influx to the point that even modest carbapenemase activity is sufficient to hydrolyze incoming drug before it reaches its targets. Thus, decreased permeability prevents carbapenemases from becoming outpaced, mirroring the inverse of our strategy in gram-positive bacteria, where palmitoleic acid-driven influx outpaces AME expression. This comparison underscores a general principle: resistance often depends on the balance between antibiotic entry and enzymatic inactivation, and adjuvants that modulate membrane permeability offer a rational path to tip that balance back in favor of the drug. Consistent with this concept, host-derived antimicrobial mechanisms can similarly restore antibiotic efficacy. For example, MAIT cell-derived cytolytic proteins increase bacterial membrane permeability, rendering carbapenem-resistant *E. coli* susceptible to carbapenem-mediated killing^39^, further supporting the idea that enhancing drug entry can overcome enzymatic resistance mechanisms.

Chronic wounds impact 2% of the United States population, with annual treatment costs exceeding $30 billion^40,41^. The majority of infected wounds are polymicrobial, with *S. aureus* and *Enterococcus spp*. being the most predominant pathogens, present in up to 93.5% and 71.7% of cases, respectively^42,43^. Biofilm formation within the wound impairs antibiotic penetration and promotes the emergence of persister and resistant cells, reducing treatment efficacy^44–50^. In addition, polymicrobial communities facilitate horizontal transfer of resistance determinants, further entrenching antimicrobial resistance in the wound environment. High-level gentamicin resistance in *E. faecalis* and *E. faecium* commonly arises through acquisition of AMEs encoded on mobile genetic elements, and these enterococci are frequently co-isolated with *S. aureus* from wound infections^51^. In addition, many of these strains are resistant to vancomycin, which is a mainstay for treating *Enterococcus* infections and is also used as a last-line agent for MRSA. Alarmingly, in diabetic patients whose wounds are co-colonized with VRE and *S. aureus*, VRE can horizontally transfer the *vanA* operon to *S. aureus*, generating VRSA^52^. In this study, we evaluated six VRE clinical isolates and one VRSA strain and found that our gentamicin-palmitoleic acid combination effectively killed mature biofilms. Together, these observations highlight how biofilm-infected wounds act as reservoirs for resistance genes, making it imperative to develop novel, rapidly acting treatment strategies that can clear these infections and curb the spread of antimicrobial resistance. Incorporating palmitoleic acid into a gentamicin topical, which is routinely used to treat gram-negative wound infections, could permit immediate treatment prior to bacterial identification, irrespective of their resistance profiles. This combination could transform a long-standing, narrow-spectrum topical antibiotic into a broadly effective agent for biofilm-infected wounds.

Palmitoleic acid is found in human serum (up to 80 µg/mL) and is generally well tolerated^53^. Palmitoleic acid oral administration has been reported to enhance metabolism and reduce insulin resistance; however, other studies associate higher circulating palmitoleic acid with increased triglyceride levels and broader metabolic disruption^54–58^. Systemic delivery is challenging as circulating palmitoleic acid is rapidly esterified into triglycerides and other lipid species, which can alter systemic lipid profiles^59,60^, while potentially reducing the pool of free fatty acid available at sites of infection. While in vitro studies have suggested potential cytotoxicity at micromolar concentrations, the physiological relevance of these findings is unclear, given the extensive protein binding of fatty acids in vivo^61,62^. Topical application of palmitoleic acid is well tolerated, with reported antibacterial, anti-inflammatory, and wound-healing properties^18^. Additionally, local administration is unlikely to substantially perturb circulating lipid homeostasis^63,64^. Notably, we did not observe evidence of enhanced toxicity with palmitoleic acid-gentamicin combination in a murine diabetic wound model^18^. Thus, local delivery of palmitoleic acid as an adjunct to gentamicin may represent a promising strategy for the treatment of chronic wound infections without substantial host toxicity.

A limitation of this combination therapy is that palmitoleic acid enhances gentamicin efficacy only against gram-positive bacteria, as it is ineffective against gram-negatives, likely because of the outer membrane^30^. Membrane-acting agents that are active against gram-negative outer membranes, such as polymyxin B^65–67^, may provide a route to extend this influx-based strategy to gram-negative pathogens^68–70^. Moreover, we also observed heterogeneity in susceptibility among gentamicin-resistant strains. Differences in baseline AME expression, as well as strain-specific efflux capacity and biofilm physiology, are likely to contribute to the variable degree of re-sensitization we observed.

We note that pre-exposure to sub-inhibitory palmitoleic acid reduces subsequent potentiation in *S. aureus* but not *E. faecalis*, suggesting that adaptive responses such as membrane remodeling may reduce drug uptake in a species-specific manner. This finding is consistent with a model in which potentiation depends on a transient window where increased antibiotic influx outpaces enzymatic inactivation. However, in vivo environments are dynamic, and prior exposure does not necessarily preclude efficacy, as we previously observed robust potentiation against a gentamicin-sensitive strain in a diabetic wound infection model^18^.

Prior efforts to overcome aminoglycoside resistance have focused on directly disabling resistance determinants, including antisense approaches to silence resistance genes, prodrug aminoglycoside-pantothenate analogs that inhibit resistance pathways in both gram-positive and gram-negative bacteria, and peptide inhibitors of AMEs^71,72^. Our work complements these strategies by using a host-compatible fatty acid to transiently tip the balance between drug influx and enzymatic inactivation. Future studies will determine whether the palmitoleic acid-gentamicin combination can resensitize gentamicin-resistant strains in vivo and whether analogous membrane-targeting adjuvants (e.g., polymyxin B for gram-negatives) can generalize this approach across diverse wound pathogens.

## Methods

### Bacterial strains and growth conditions

**Table:** 

Strains	Identifier	Genotype or relevant characteristics	Source
***S. aureus***			
JE2	BC2127	Wild type MRSA strain	^73^
HIP11714	BC2069	VRS1, isolated from patient exit catheter site in 2002, NR-46410	BEI Resources, NIAID, NIH^62,74^
	BC329	Isolated from cystic fibrosis patient	^18^
RN4220	BC6	Wild type MSSA strain	^62^
***E. faecalis***			
OG1RF	BC2015	Wild type strain	^18^
SF28073	BC2064	Isolated from patient urine sample in 2003, NR-31972	BEI Resources, NIAID, NIH^74^
TX0104	BC2065	Isolated from patient blood sample in 2002, HM-201	BEI Resources, NIAID, NIH^62,74^
V587	BC2067	EnGen0242, isolated from patient urine sample in 1987, NR-31979	BEI Resources, NIAID, NIH^62,74^
***E. faecium***			
TX0082	BC2063	Isolated from patient blood sample in 1999, HM-460	BEI Resources, NIAID, NIH^74^
UAA714	BC2066	EnGen0158, isolated in 1994, NR-32065	BEI Resources, NIAID, NIH^74^
Patient #2-1	BC2068	EnGen0316, isolated from patient stool sample NR-31909	BEI Resources, NIAID, NIH^74^
***E. coli***			
BL21(DE3)		C2528H	New England Biolabs

*S. aureus* isolates (JE2^73^, VRS1^62,74^, BC329^18^) were grown in Tryptic Soy Broth (TSB) (Remel) at 37 °C with continuous shaking at 225 rpm. *E. faecalis* (OG1RF^18^, SF28073^74^, TX0104^62,74^, V587^62,74^) and *E. faecium* isolates (TX0082^74^, UAA714^74^, BC2068^74^) were grown in Brain Heart Infusion (BHI) media (Oxoid) at 37 °C statically.

### Minimum inhibitory concentration assay

Minimum inhibitory concentration assays (MICs) of gentamicin (Fisher Bioreagents) ± 15 µg/mL palmitoleic acid (Cayman Chemical), as well as MICs for palmitoleic acid alone, were determined using the broth microdilution method. *S. aureus* isolates (JE2, VRS1, BC329) were grown overnight (18 h) in TSB (Remel), and *E. faecalis* (OG1RF, SF28073, TX0104, V587) and *E. faecium* isolates (TX0082, UAA714, BC2068) were grown in BHI (Oxoid). Each isolate was diluted in Mueller–Hinton Broth (MHB) (Oxoid) or BHI. Approximately 5 × 10^5^ CFU/well was incubated with various concentrations of drug in a 96-well plate (Corning). The plate was covered with a Breathe Easier sealing membrane (Sigma) and incubated at 37 °C for 18–20 h. The MIC was determined at the lowest concentration of gentamicin that prevented visible growth. Gentamicin ± palmitoleic acid MICs were performed in triplicate on separate days. Palmitoleic acid MICs were performed in duplicate on separate days. Bar graphs show the median from nine biological replicates across three independent experiments. Gentamicin sulfate is soluble at concentrations up to 250 mg/mL. Accordingly, the highest concentration evaluated in this study was 125 mg/mL, as antimicrobial susceptibility assays involve a two-fold dilution upon addition of the bacterial inoculum, resulting in a final maximum concentration of 125 mg/mL in the assay.

### Checkerboard assay

Fractional inhibitory concentration (FIC) assays of gentamicin and palmitoleic acid were determined using the broth microdilution method. *S. aureus* isolates (JE2, VRS1) and *E. faecalis* OG1RF were grown overnight (18 h) in TSB (Remel) and BHI (Oxoid), respectively. Each isolate was diluted in MHB (Oxoid). Approximately 5 × 10^5^ CFU was incubated with increasing concentrations of drug in a 96-well plate (Corning). The plate was covered with a Breathe Easier sealing membrane (Sigma) and incubated at 37 °C for 18–20 h. OD_600_ was measured, and the FIC index was calculated as follows: concentration ^PA^/MIC^PA^ + concentration ^gent^/MIC^gent^ = FIC^PA^ + FIC^gent^ = FICI. An FIC value of ≤0.5 indicates synergy^75^.

### Biofilm assays

Bacteria were cultured overnight (18 h) in TSB (Remel) (*S. aureus* JE2, VRS1, BC329) or BHI (Oxoid) (*E. faecalis* OG1RF, SF28073, TX0104, V587, and *E. faecium* TX0082, UAA714, BC2068) in biological triplicates. Each culture was diluted 1:150 in BHI and added to a 96-well tissue culture-treated plate (Costar). Plates were covered with a Breathe Easier sealing membrane (Sigma) and incubated at 37 °C for 24 h. Biofilms were washed twice with PBS before the addition of fresh BHI with 100–1000 µg/mL gentamicin (Fisher Bioreagents) ± 30 µg/mL palmitoleic acid (Cayman Chemical). Biofilms were covered and incubated at 37 °C for 24 h. Biofilms were washed twice with PBS, then dispersed via water bath sonication and vigorous pipetting. Cells were enumerated by serial dilution in PBS and plating. Assays were performed in duplicate or triplicate, as indicated, on separate days. Higher concentrations of palmitoleic acid (30 μg/mL) were used in the biofilm assays than in the MIC assays to account for the substantially higher bacterial density present in biofilms (~10^8^ CFU/mL) compared to standard MIC assays (~10^6^ CFU/mL). For experiments involving inducible expression constructs, 0.2% xylose (Fisher BioReagents) was added 24 h prior to antibiotic treatment to induce protein production. Strains harboring pEPSA5::*gfp*^76^ were used as controls, as heterologous protein expression can impose a metabolic burden that can decrease antibiotic susceptibility, as reported previously^77^. In spent media experiments, wash steps prior to antibiotic treatment and addition of fresh BHI were omitted, and 100 µg/mL gentamicin and 30 µg/mL palmitoleic acid were added directly to the biofilms. For pre-exposure experiments, diluted cultures were treated with 7.5 µg/mL palmitoleic acid for 24 h prior to antibiotic exposure.

### Flow cytometry

Bacteria were cultured overnight (18 h) in TSB (Remel) (*S. aureus* JE2, VRS1, BC329) or BHI (Oxoid) (*E. faecalis* OG1RF, SF28073, TX0104, V587, and *E. faecium* TX0082, BC2068) in biological triplicate. Each culture was diluted 1:150 in MHB and added to a 96-well tissue culture-treated plate (Costar). Biofilms were covered with a Breathe Easier sealing membrane (Sigma) and incubated at 37 °C for 24 h. Biofilms were washed twice with PBS. Gentamicin (Fisher Bioreagents) was conjugated to Texas Red-succinimidyl ester (Invitrogen) as previously described^18^. Fresh BHI with 100 µg/mL gentamicin conjugated to Texas Red ± 30 µg/mL palmitoleic acid (Cayman Chemical) were added to the biofilms, and the biofilms were covered and incubated at 37 °C for 3–5 h. Biofilms were washed twice with PBS and then dispersed via vigorous pipetting. Effective disruption of aggregates was confirmed by microscopy. Samples were diluted in 1% saline (Fisher Bioreagents) in polystyrene tubes (Falcon). Texas Red gentamicin uptake was detected on the LSRII Fortessa (BD Biosciences) with 50,000 events collected. Bar graphs show means ± SD from six biological replicates across independent experiments or three biological replicates in a representative experiment, as indicated; experiments were independently repeated on ≥2 separate days with similar results.

### Construction of bifunctional aminoglycoside modifying enzyme (AME) expression plasmids

#### To create an inactive gentamicin standard for HRMS

For the construction of *pBAD33-aac(6’)-Ie-aph(2”)-Ia* (CLON68), *aac(6’)-Ie-aph(2”)-Ia*, and a histidine tag at the N-terminus was PCR amplified from a gBlock (Integrated DNA Technologies) using primers 5’-CTCCATACCCGTTTTTTTGGGCTAGCGAATTCGAGCTCGG-3’ and 5’-CAGGCTGAAAATCTTCTCTCATCCGCCAAAACAGCCAAG-3’. Inverse PCR was performed to linearize *pBAD33* downstream of the *araBAD* promoter using primers 5’-CTTGGCTGTTTTGGCGGATGAGAGAAGATTTTCAGCCTGA-3’ and 5’-CCGAGCTCGAATTCGCTAGCCCAAAAAAACGGGTATGGAG-3’. The *aac(6’)-Ie-aph(2”)-Ia* was inserted downstream of the *araBAD* promoter on the *pBAD33* plasmid using NEBuilder HiFi DNA Assembly (New England Biolabs). The plasmid was transformed into *Escherichia coli* BL21. Transformed bacterial colonies were selected on Luria-Bertani (Miller) agar plates containing 10 µg/mL chloramphenicol and were confirmed via sequencing.

#### For the construction of the AAC-APH overexpression plasmid *pEPSA5-aac(6’)-Ie-aph(2”)-Ia* (CLON70)

*aac(6’)-Ie-aph(2”)-Ia* was PCR amplified from a gBlock containing the codon-optimized gene from *E. faecalis* OG1RF (Integrated DNA Technologies) using primers 5’-TCTAGAGGATCCCCGGGTACTCAGTGATGGTGGTGATGGT-3’ and 5’-ATTAACCAACTAAAATGTAGATGAATATCGTAGAAAACGAGATTTG-3’. The *pEPSA5* backbone was linearized via double digestion with EcoRI-HF and KpnI-HF (New England Biolabs), and the plasmid was assembled using NEBuilder HiFi DNA Assembly (New England Biolabs). Plasmids were transformed initially into restriction site-deficient *S. aureus* RN4220 and then into *S. aureus* JE2 via electroporation as previously described^78,79^.

### AAC-APH protein production and purification

*E. coli* BL21(DE3) harboring the *pBAD33-aac(6’)-Ie-aph(2”)-Ia* plasmid was grown overnight in LB with 10 µg/mL chloramphenicol. The culture was diluted in fresh media and grown at 37 °C with shaking at 225 rpm for 2 h. 2% arabinose was added to the culture to induce protein production and protein production occurred overnight (16–18 h) at 16 °C with shaking at 225 rpm. Cells were pelleted and resuspended in 25 mM HEPES (Fisher Bioreagents), 200 mM NaCl buffer. The cells were lysed via a French press, and the supernatant was run through a Ni-NTA resin (Qiagen). The column was washed with 50 mM imidazole (Acros Organics), 25 mM HEPES, 200 mM NaCl, pH 7.5. The protein was eluted using increasing concentrations of imidazole (100-500 mM) in a 25 mM HEPES and 200 mM NaCl buffer. The eluted protein was further purified via size exclusion chromatography. The protein was concentrated using a 10 K MWCO Amicon filter (Millipore) prior to application to a Superdex 200 column equilibrated in 25 mM HEPES (Fisher Bioreagents) with 200 mM sodium chloride (Fisher Bioreagents) using a GE AKTA chromatography system and confirmed via SDS-PAGE (Invitrogen) (Supplementary Fig. 4B). Protein concentration was estimated using Abs_280_ using the calculated extinction coefficient (46,980 M^−1^cm^−1^). Protein was stored in PBS at −80 °C.

### Confirmation of AAC-APH activity

Minimum inhibitory concentration assays (MICs) of gentamicin (Fisher Bioreagents) ± exogenous AAC-APH were determined using the broth microdilution method (Supplementary Fig. 4C). *S. aureus* JE2 was grown overnight (18 h) in TSB (Remel). The culture was diluted in MHB (Oxoid). Approximately 5 × 10^5^ CFU/well was incubated with various concentrations of gentamicin with and without 400 nM purified histidine-tagged AAC-APH and protein co-factors 100 µM acetyl-coenzyme A (Cayman Chemical), 100 µM guanosine 5’-triphosphate (Cayman Chemical), and 250 µM MgCl_2_ (Mallinckrodt) in a 96-well plate (Corning). The plate was covered with a Breathe Easier sealing membrane (Sigma) and incubated at 37 °C for 18–20 h. The MIC was determined at the lowest concentration of gentamicin that prevented visible growth.

### High-resolution mass spectrometry (HRMS)

#### To prepare an inactive gentamicin standard for HRMS

An inactive gentamicin reference was generated by incubating gentamicin with purified AAC(6’)-Ie-APH(2”)-Ia under cell-free conditions, enabling confident assignment of enzymatically modified gentamicin species detected by HRMS. Briefly, 500 nM purified AAC-APH was combined with 100 µM acetyl-coenzyme A (Cayman Chemical), 100 µM guanosine 5’-triphosphate (Cayman Chemical), 250 µM MgCl_2_ (Mallinckrodt), and 16 µg/mL gentamicin. The reaction was incubated at room temperature for 18 h before being analyzed by the UNC Center for AIDS Research (CFAR) Core.

#### To prepare samples for analysis by HRMS

*S. aureus* JE2 and VRS1 were grown overnight (16–18 h) in TSB. The cultures were diluted 1:100 into fresh media and grown to mid-exponential phase at 37 °C with shaking at 225 rpm. Cultures were treated with gentamicin at 5x the MIC ± 15 µg/mL palmitoleic acid, and at 5 h post-treatment, surviving cells were pelleted, washed with PBS to remove extracellular drugs, and lysed with 10 mg/mL lysostaphin (Ambi Products), and the reaction was incubated at 37 °C for 10 min. Once the cells were lysed, the lysostaphin was heat-inactivated at 99 °C for 5 min. Lysates were stored at −20 °C until analyzed by the CFAR Core.

#### To characterize samples by HRMS

Gentamicin samples were analyzed on a Q Exactive Plus Orbitrap mass spectrometer (Thermo Scientific) equipped with a HESI ion source using direct infusion. The Orbitrap analyzer was operated in positive ion mode (electrospray voltage: 3.5 kV; capillary temperature: 300 °C; sheath gas flow rate: 6; S Lens RF level: 50). Data acquisition was conducted by Full MS acquisition by 1 microscan with mass resolution of 140,000 (at m/z 200) and automatic gain control (AGC) target of 1 × 10^6^. A pure gentamicin standard was diluted to 1 µg/mL in electrospray solution (50% methanol, 49.8% water, 0.2% formic acid). Samples prepared in PBS were diluted 10-fold in the same electrospray solution. Collected mass spectra (*n* = 50) from each sample were processed using FreeStyle (Thermo Scientific).

## Supplementary information

Supplementary information

## Data Availability

Raw data used to generate figures is available in a persistent repository (UNC Dataverse).
