# Supplementary material for: Palmitoleic acid drives gentamicin influx to outpace aminoglycoside-modifying enzymes and overcome resistance
Source: NPJ Antimicrob Resist. 2026 Jul 14;4:75. doi: 10.1038/s44259-026-00243-2 (PMC13745537; doi:10.1038/s44259-026-00243-2)
Supplement: Supplementary file 1 [file 44259_2026_243_MOESM1_ESM.docx]

**SUPPLEMENTARY MATERIALS**

**Supplementary Table 1**. **Palmitoleic acid and aminoglycoside MICs.**

| **Strains** | **Palmitoleic acid MIC (µg/mL)** | **Kanamycin MIC (µg/mL)** | **Kanamycin palmitoleic acid MIC (µg/mL)** | **Amikacin MIC (µg/mL)** | **Amikacin palmitoleic acid MIC (µg/mL)** |
| --- | --- | --- | --- | --- | --- |
| *S. aureus* JE2 | 32 |  |  |  |  |
| *S. aureus* VRS1 | 32 | >25,000 | 48 | 6250 | 4 |
| *S. aureus* BC329 | 32 |  |  |  |  |
| *E. faecalis* OG1RF | 32 |  |  |  |  |
| *E. faecium* TX0082 | 32 |  |  |  |  |
| *E. faecalis* SF28073 | 32 |  |  |  |  |
| *E. faecalis* TX0104 | 32 |  |  |  |  |
| *E. faecium* UAA714 | 32 |  |  |  |  |
| *E. faecalis* V587 | 32 |  |  |  |  |
| *E. faecium* BC2068 | 32 |  |  |  |  |

**Supplementary Table 2. High-resolution mass spectrometry signal abundance and frequency.**

|  |  | **Gentamicin Standard** | | **Modified Gentamicin** | | **JE2 gentamicin** | | | | **VRS1 gentamicin** | | | | **VRS1 gentamicin + palmitoleic acid** | | | |
| --- | --- | --- | --- | --- | --- | --- | --- | --- | --- | --- | --- | --- | --- | --- | --- | --- | --- |
|  |  |  |  |  |  | **Replicate 1** | | **Replicate 2** | | **Replicate 1** | | **Replicate 2** | | **Replicate 1** | | **Replicate 2** | |
|  | ***(m/z)*** | **Signal Abundance** | **Frequency** | **Signal Abundance** | **Frequency** | **Signal Abundance** | **Frequency** | **Signal Abundance** | **Frequency** | **Signal Abundance** | **Frequency** | **Signal Abundance** | **Frequency** | **Signal Abundance** | **Frequency** | **Signal Abundance** | **Frequency** |
| **C1** | 239.665 | 2.26E+07 | 100% | 4.93E+02 | 12% | 1.30E+05 | 100% | 4.93E+04 | 98% | 4.87E+04 | 96% | 2.89E+03 | 64% | 2.50E+04 | 100% | 1.30E+04 | 99% |
| **C2** | 232.658 | 1.36E+07 | 100% | 0.00E+00 | 0% | 6.03E+03 | 96% | 1.32E+03 | 38% | 1.29E+04 | 92% | 6.08E+01 | 3% | 5.94E+03 | 100% | 7.93E+02 | 31% |
| **C1a** | 225.650 | 2.73E+06 | 100% | 0.00E+00 | 0% | 4.54E+02 | 12% | 0.00E+00 | 0% | 2.89E+02 | 15% | 0.00E+00 | 0% | 9.16E+01 | 9% | 2.52E+01 | 1% |
| **Ac C1** | 260.671 | 2.73E+02 | 2% | 0.00E+00 | 0% | 0.00E+00 | 0% | 0.00E+00 | 0% | 0.00E+00 | 0% | 0.00E+00 | 0% | 7.56E+01 | 4% | 0.00E+00 | 0% |
| **Ac C2** | 253.663 | 5.01E+04 | 96% | 4.32E+04 | 100% | 0.00E+00 | 0% | 0.00E+00 | 0% | 0.00E+00 | 0% | 0.00E+00 | 0% | 1.10E+03 | 55% | 0.00E+00 | 0% |
| **Ac C1a** | 246.655 | 4.28E+04 | 98% | 3.03E+04 | 100% | 0.00E+00 | 0% | 0.00E+00 | 0% | 0.00E+00 | 0% | 0.00E+00 | 0% | 2.67E+03 | 94% | 0.00E+00 | 0% |
| **Phos C1** | 279.649 | 0.00E+00 | 0% | 1.02E+05 | 100% | 0.00E+00 | 0% | 0.00E+00 | 0% | 6.15E+03 | 88% | 3.81E+03 | 78% | 1.72E+05 | 100% | 5.43E+03 | 82% |
| **Phos C2** | 272.641 | 0.00E+00 | 0% | 3.93E+04 | 100% | 0.00E+00 | 0% | 0.00E+00 | 0% | 7.47E+02 | 24% | 1.58E+02 | 8% | 4.90E+04 | 100% | 4.94E+02 | 14% |
| **Phos C1a** | 265.633 | 0.00E+00 | 0% | 0.00E+00 | 0% | 0.00E+00 | 0% | 0.00E+00 | 0% | 0.00E+00 | 0% | 1.72E+01 | 1% | 6.45E+02 | 35% | 0.00E+00 | 0% |
| **Phos ac C1** | 300.654 | 0.00E+00 | 0% | 0.00E+00 | 0% | 0.00E+00 | 0% | 0.00E+00 | 0% | 4.71E+01 | 2% | 1.04E+02 | 4% | 4.44E+03 | 96% | 3.16E+02 | 9% |
| **Phos ac C2** | 293.646 | 0.00E+00 | 0% | 5.56E+04 | 100% | 0.00E+00 | 0% | 0.00E+00 | 0% | 1.10E+04 | 98% | 1.04E+02 | 4% | 2.35E+05 | 98% | 2.95E+04 | 96% |
| **Phos ac C1a** | 286.638 | 0.00E+00 | 0% | 3.48E+04 | 100% | 0.00E+00 | 0% | 0.00E+00 | 0% | 6.64E+03 | 92% | 9.31E+03 | 94% | 1.35E+05 | 98% | 8.12E+03 | 79% |

**Supplementary Figure 1. Palmitoleic acid and gentamicin display synergy against gentamicin-resistant strains. (A)** *S. aureus* JE2, **(B)** *S. aureus* VRS1, and **(C)** *E. faecalis* OG1RF were incubated with increasing concentrations of gentamicin and palmitoleic acid. OD_600_ was measured and shown. White wells indicate no growth and gray wells indicate growth. Experiments were independently repeated on ≥2 separate days with similar results.

**Supplementary Figure 2.** **Palmitoleic acid-mediated potentiation of gentamicin in biofilms is reduced under nutrient-depleted conditions.** Mature biofilms from *S. aureus* strains **(A)** JE2 or **(B)** VRS1 or **(C)** *E. faecalis* OG1RF were cultured for 24h in BHI media prior to treatment with 100 µg/mL gentamicin (gent) ± 30 µg/mL palmitoleic acid (PA) (without adding fresh media). Survivors were enumerated after 24h. Bar graphs show means ± SD from n=6 biological replicates in a representative experiment; experiments were independently repeated on 2 separate days with similar results. Statistical significance was determined using an unpaired t-test. ns, *, and **** denotes not significant, p < 0.05 and p < 0.0001, respectively.

**Supplementary Figure 3.** **Pre-exposure to sub-MIC palmitoleic acid reduces subsequent palmitoleic acid-mediated potentiation of gentamicin in *S. aureus* but not *E. faecalis*.** Biofilms from **(A)** *S. aureus* VRS1 or **(B)** *E. faecalis* OG1RF were cultured for 24h in BHI media containing 7.5 µg/mL palmitoleic acid prior to treatment with 100 µg/mL gentamicin (gent) ± 30 µg/mL palmitoleic acid (PA). Survivors were enumerated after 24h. Bar graphs show means ± SD from n=6 biological replicates in a representative experiment; experiments were independently repeated on 2 separate days with similar results. Statistical significance was determined using an unpaired t-test. ns and **** denotes not significant and p < 0.0001, respectively.

**Supplementary Figure 4. Purifying an AME to generate a modified gentamicin standard for HRMS** **(A)** AlphaFold^79^ prediction of the AAC(6’)-Ie-APH(2’’)-Ia, with the acetyltransferase (AAC) in blue at the N-terminus and the phosphotransferase (APH) in red at the C-terminus. **(B)** SDS-PAGE of the purified histidine-tagged AAC(6’)-Ie-APH(2’’)-Ia at each elution step with increasing concentrations of imidazole with desired protein product at 56 kDa. **(C)** *S. aureus* JE2 gentamicin MIC with and without the presence of 400 nM AAC-APH.
